# Division of labour in colony defence in a clonal ant

**DOI:** 10.1098/rstb.2023.0270

**Published:** 2025-03-20

**Authors:** Zimai Li, Qi Wang, Daniel Knebel, Daniel Veit, Yuko Ulrich

**Affiliations:** ^1^ Max Planck Institute for Chemical Ecology, Jena 07745, Germany; ^2^ Faculty of Biological Sciences, Friedrich Schiller University Jena, Jena 07743, Germany; ^3^ Max Planck Institute for the History of Science, Berlin 14195, Germany

**Keywords:** division of labour, automated tracking, clonal raider ant, colony defence

## Abstract

Division of labour (DOL) plays a key role across all scales of biological organization, but how its expression varies across contexts is still poorly understood. Here, we measure DOL in a crucial task, colony defence, in a social insect that affords precise experimental control over individual and colony traits, the clonal raider ant (*Ooceraea biroi*). We find that DOL in defence behaviour emerges within colonies of near-identical workers, likely reflecting variation in individual response thresholds, and that it increases with colony size. Additionally, colonies with pupae show higher defence levels than those without brood. However, we do not find evidence for a behavioural syndrome linking defence with exploration and activity, as previously reported in other systems. By showing how colony composition and size affect group response to potential threats, our findings highlight the role of the social context in shaping DOL.

This article is part of the theme issue ‘Division of labour as key driver of social evolution’.

## 1. Introduction

Division of labour (DOL), whereby members of a group specialize in distinct tasks, is a key feature of social systems from microorganisms [1] to insects [2,3] and humans [4]. DOL plays a key role at all scales of biological organization [5] and the emergence of new forms of DOL underlies all the major evolutionary transitions, such as those from prokaryotic to eukaryotic cells, from unicellular to multicellular organisms and from solitary to eusocial life [6–8]. DOL often increases with within-group heterogeneity, e.g. in age [2,9], genetic background [10,11] or morphology [12], which is believed to create the conditions necessary for individuals to specialize in different tasks.

Social insects (ants, termites, bees and wasps) are among the most ecologically successful animals [13,14] and display some of the most extreme and elaborate forms of DOL [13,15,16]. Typical social insect colonies have reproductive DOL between one or a few queens, which monopolize reproduction, and (functionally) sterile workers, which perform all other tasks needed for colony maintenance [17]. Additionally, there is (non-reproductive) DOL among the workers, which specialize in a subset of maintenance tasks (e.g. foraging, nursing, defence). Thus, like a multicellular organism, a social insect colony is composed of related units (individuals in insect colonies, cells in multicellular organisms) that are specialized in different essential tasks, like reproduction (queens in social insect colonies, germline cells in multicellular organisms) or energy storage (repletes in some insect colonies, adipocytes in multicellular organisms) and therefore depend on each other for survival. As a consequence, social insect colonies are often conceptualized as ‘superorganisms’ [18].

A striking example of specialization in social insect workers is in colony defence. Most social insects live in stable nests, which must be defended against various external threats, including conspecific and allospecific intruders and competitors, predators and parasites [19–21]. Analogous to the immune cells that specialize in defending multicellular organisms against external threats like pathogens, some members of social insect colonies often specialize in colony defence [22,23]. The best-documented and most iconic cases are the morphologically specialized soldier castes of some termites [24], ants [25] and bees [26]. Age polyethism in defence has also been reported, whereby older workers engage in defence more readily than young workers [12,27,28]. However, it remains unclear whether DOL in defence can emerge in the absence of inter-individual differences in morphology or age.

In social insects and other animals, inter-individual variation in aggressive behaviour has often been described as part of a ‘behavioural syndrome’ linking exploration, aggression and boldness across individuals [29–33]. For example, in great tits (*Parus major*), juvenile males that are more exploratory are also more aggressive (initiate more fights with conspecifics) [34]. Similarly, individuals belonging to the patroller caste of *Myrmica* ants show higher aggression towards allospecifics, increased exploration of a novel environment and higher activity in response to alarm pheromones [35]. Empirical studies on behavioural syndromes in social insects typically measure behavioural traits in isolated individuals (i.e. outside the colony context). Studies conducted in a social setting are rare and results are equivocal [36–38]. Furthermore, individual variation in exploration and defence behaviours is often associated with variation in age and genetic background [28,39–41], and it remains unclear whether behavioural syndromes manifest among workers of social insect colonies when these factors are controlled [29].

Here, we investigate DOL in colony defence against allospecific intruders in the clonal raider ant, *Ooceraea biroi*. In this species, colonies are queenless and composed of workers that reproduce asexually and synchronously, so that genetically near-identical adults are produced in discrete age cohorts [42]. This allows us to investigate DOL in colony defence in the absence of variation in age or genetic background between individuals and between colonies. We first ask whether there is DOL in colony defence. Then, we test whether two aspects of the social environment, colony size and brood treatment, affect DOL and efficiency in colony defence. Group size has been shown to increase DOL in other tasks (intranidal versus extranidal tasks) in the clonal raider ant [43]. We expected the presence and type of brood to affect DOL in defence in the clonal raider ant because the brood is known to affect worker physiology and behaviour in this species [42,44]. Finally, we ask whether DOL in colony defence is linked to a behavioural syndrome by testing for an association between individual exploratory behaviour, activity and aggressive behaviour in a colony defence context.

## 2. Methods

### (a) Experimental design

Clonally related, age-matched (77-day-old) ants of clonal lineage B [45] were randomly selected from one stock colony, individually marked on the thorax and gaster using oil-paint markers (Uni Paint PX-20 and PX-21) and used to form experimental colonies with two size treatments (4 workers or 8 workers) and three brood treatments: no brood, larvae (as many 5-day-old larvae as there were workers) or pupae (as many 6-day-old yellow pupae as there were workers). Six replicate colonies were used for each of the six treatments.

**Figure 1.**
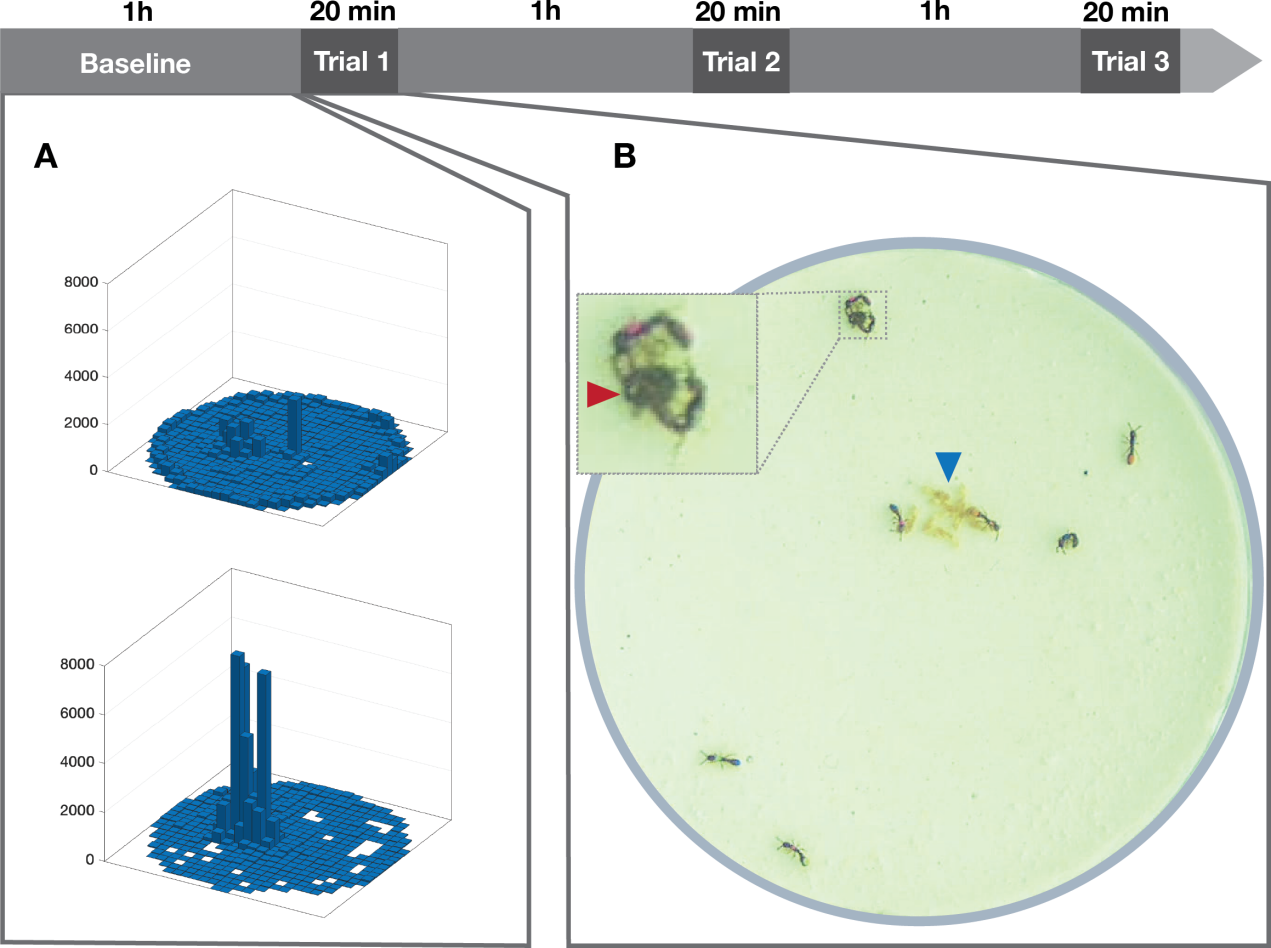
Experimental procedure. (*a*) Three-dimensional histograms of the spatial distribution of one ant with high entropy (top: *H* = 8.415) and one ant from the same colony with lower entropy (bottom: *H* = 3.041) at baseline. The *z*-axis represents the number of frames in which the ant occupied each grid cell. (*b*) Experimental ant colony in a colony defence trial; blue arrowhead: brood pile (pupae). Inset: zoomed-in view of defence behaviour by two clonal raider ants towards an intruder (red arrowhead).

Experimental colonies were housed in airtight Petri dishes 5 cm in diameter, with a plaster of Paris floor saturated with water. *Ooceraea biroi* feeds on the brood of other ant species [42,46], and colonies were fed *Tetramorium bicarinatum* pupae proportionally to group size (two pupae for colonies of size 4, and four pupae for those of size 8) once. Two days after the colonies were established, we recorded colony behaviour at 20 frames per second (fp)s using 6 Basler (model acA20440-20gc; Ahrensburg, Germany) cameras and LoopBio Motif (v. 6) software. We first recorded 1 h of colony baseline activity (figure 1A), followed by three trials of colony defence at 1 h intervals. In each trial, we introduced a dead (freeze-killed and thawed) worker of *T. bicarinatum* as an allospecific ‘intruder’ in each colony and recorded a 30 min video. Pilot experiments were conducted to ensure that the intruder was perceived as an external threat and not as food. Results of the pilots showed that *O. biroi* workers attacked dead workers but not the brood of *T. bicarinatum* and that dead workers were not consumed, while the brood was. The use of a dead intruder allowed us to measure colony behavioural responses while ruling out any effects of the intruder’s behaviour [47,48]. Intruders were removed after each trial, and a fresh intruder was used in each trial. All experiments were conducted in a climate chamber maintained at 28.20 ± 0.20°C.

### (b) Behavioural data acquisition

Baseline activity in the first hour of recording (before defence trials) was analysed using automated tracking with anTraX [49]. This detected the position of each ant every 50 ms. Raw tracking data underwent preprocessing in MATLAB (R2023b) to identify missing or aberrant positions, which were then interpolated or removed, respectively, following [50].

In each defence trial, we used BORIS [51] to manually annotate behaviour in the first 20 min after the introduction of the intruder. We annotated encounters of all clonal raider ants (individually identifiable by their unique paint marks) with the intruder as well as their defence behaviour towards the intruder. We quantified the number of encounters, defined as physical contacts with the intruder, as well as the number and duration of stinging attempts (in seconds), defined by a stereotyped holding of the intruder with the mouthparts and bending of the gaster towards the intruder (figure 1B; [52]).

### (c) Behavioural data analysis

Unless stated otherwise, analyses were conducted using R v. 4.3.1 [53].

To measure individual baseline exploratory behaviour, we calculated the entropy of individual spatial distribution in the first hour of recording, before defence trials [54]. To this aim, the area of each Petri dish was binned (25 × 25 bins of 2 × 2 mm each; 2 mm is approximately one ant body length). For each ant, we calculated Shannon entropy: H = −∑ p(x) logp(x) , where *p*(*x*) is the proportion of frames that an ant spent in bin *x*. Higher entropy corresponds to movement patterns that are more evenly distributed across the grid (indicative of higher exploratory behaviour), while lower entropy values correspond to movement patterns that are more restricted to certain areas of the grid (indicative of lower exploratory behaviour; figure 1A). To measure individual baseline activity, for each ant, we calculated: (i) the proportion of time active, defined as the proportion of frames in which an ant was moving at a speed greater than 1 mm s⁻¹; and (ii) the mean duration of active bouts, defined as the mean number of consecutive frames of activity, which represents how long an ant stays active during each period of activity.

To measure an individual’s propensity to engage in defence, for each ant, we calculated a defence score as:


$$\frac{\;number\;of\;stinging\;attempts}{\;number\;of\;encounters}\;\times log\left(1\;+\;duration\;of\;stinging\;attempts\right).$$

The score incorporates both the number and duration of an individual’s stinging attempts and uses the logarithm of stinging attempt duration to prevent long stinging attempts from inflating the defence score [47]. The number of stinging attempts is normalized by the number of encounters to capture the individual propensity to engage in defence behaviour upon encountering an intruder, i.e. to correct for variation in encounter rates (which might arise from, e.g. variation in locomotor activity). The defence score takes the value 0 for ants that never stung the intruder and was assigned the value 0 for ants that never encountered the intruder [55]. The defence score correlates with the simpler normalized number of stinging attempts (number of stinging attempts/number of encounters; electronic supplementary material, figure S1), and statistical analyses using either metric yielded qualitatively similar results (electronic supplementary material, tables S1–S4). We calculated the individual defence score of each ant within each trial as well as across all three trials.

To measure DOL in colony defence, we quantified within-individual consistency and between-individual variation in defence behaviour as in [43]. Individual consistency in defence behaviour between trials was calculated by ranking individual defence scores within each colony in each trial (assigning the same lowest rank to ties) and conducting Spearman’s rank correlation tests between successive trials (Trial 1 versus Trial 2 and Trial 2 versus Trial 3) for each group size and (separately) for each brood treatment. *p*-values from the correlation tests were adjusted using the Benjamini–Hochberg procedure to correct for multiple comparisons. Colonies that did not show defence behaviour in either trial of each pair of trials were excluded from the analysis. To quantify within-colony variation in defence behaviour, we calculated the standard deviation of individual defence scores for all ants in a colony, in each trial separately as well as across all three trials. To investigate factors influencing behavioural variation in colony defence, we used a Gaussian generalized linear mixed model (GLMM, *lmer* function from package *lme4*) to analyse the effects of brood treatment (a three-level factor), group size (a two-level factor), trial (a three-level factor) and colony mean defence score (to account for overall defensive effort), as well as all their interactions, on behavioural variation, with colony as a random effect. We used the *drop1* function (package *stats*) to evaluate the significance of terms and to reduce models by iteratively deleting non-significant interactions. We validated model assumptions using the *simulateResiduals* function from the *DHARMa* package. Additionally, to assess the impact of group size on behavioural variation while avoiding artefacts arising from sampling effects, we employed a resampling approach following [43]. We simulated colonies of size 4 by randomly selecting 4 individuals (without replacement) from each colony of size 8. Behavioural variation was calculated for each simulated colony and averaged across replicate simulated colonies. This resampling procedure was repeated 1000 times. To evaluate whether the behavioural variation of colonies of size 4 significantly differed from that of size 8, we generated 95% confidence intervals for the behavioural variation of simulated colonies using the resampled data.

For each colony, defence efficiency was defined as the total number of stinging attempts received by the intruder across all trials. This is based on the assumption that a higher number of stings is more likely to lead to the retreat or death of intruders and therefore represents a more efficient defence [56]. We assessed the effects of group size (a two-level factor) and brood treatment (a three-level factor), as well as their interactions, on colony defence efficiency using a linear regression model (LM, *lm* function from package *stats*). We used the *drop1* function to evaluate the significance of terms and to reduce models by iteratively deleting non-significant interactions. We then performed pairwise comparisons between the levels of significant factors, using Tukey’s *post hoc* tests (function *emmeans* from package *emmeans*). Model assumptions of the LM were validated with the diagnostic plots produced by the *autoplot* function from package *ggfortify*.

To examine the relationship between individual baseline exploratory behaviour, activity and defence behaviour, we tested whether (i) exploratory behaviour affected encounters with the intruder—as would be expected if ants randomly encountered the intruder while patrolling their environment—using a Gaussian generalized linear mixed model (GLMM; function *glmmTMB* from the *glmmTMB* package), with the number of encounters in trial 1 (a continuous variable) as response variable, individual entropy (a continuous variable), brood treatment (a three-level factor) and colony size (a two-level factor), as well as all their interactions, as predictors, and colony as a random effect. We used the *drop1* function to evaluate the significance of terms and to reduce models by iteratively deleting non-significant interactions. (ii) The two measures of activity (proportion of time active and mean duration of active bouts, both continuous variables) correlated with each other and with entropy using Spearman’s rank correlation tests; (iii) individual exploratory behaviour and activity affected defence scores using separate Gaussian GLMMs for each predictor (individual entropy, proportion of time active and mean duration of active bouts), with brood treatment (a three-level factor) and colony size (a two-level factor) as additional predictors, colony as a random effect, and individual defence score in trial 1 as the response variable. We validated the assumptions of all GLMMs using the *simulateResiduals* function from the *DHARMa* package.

## 3. Results

**Figure 2.**
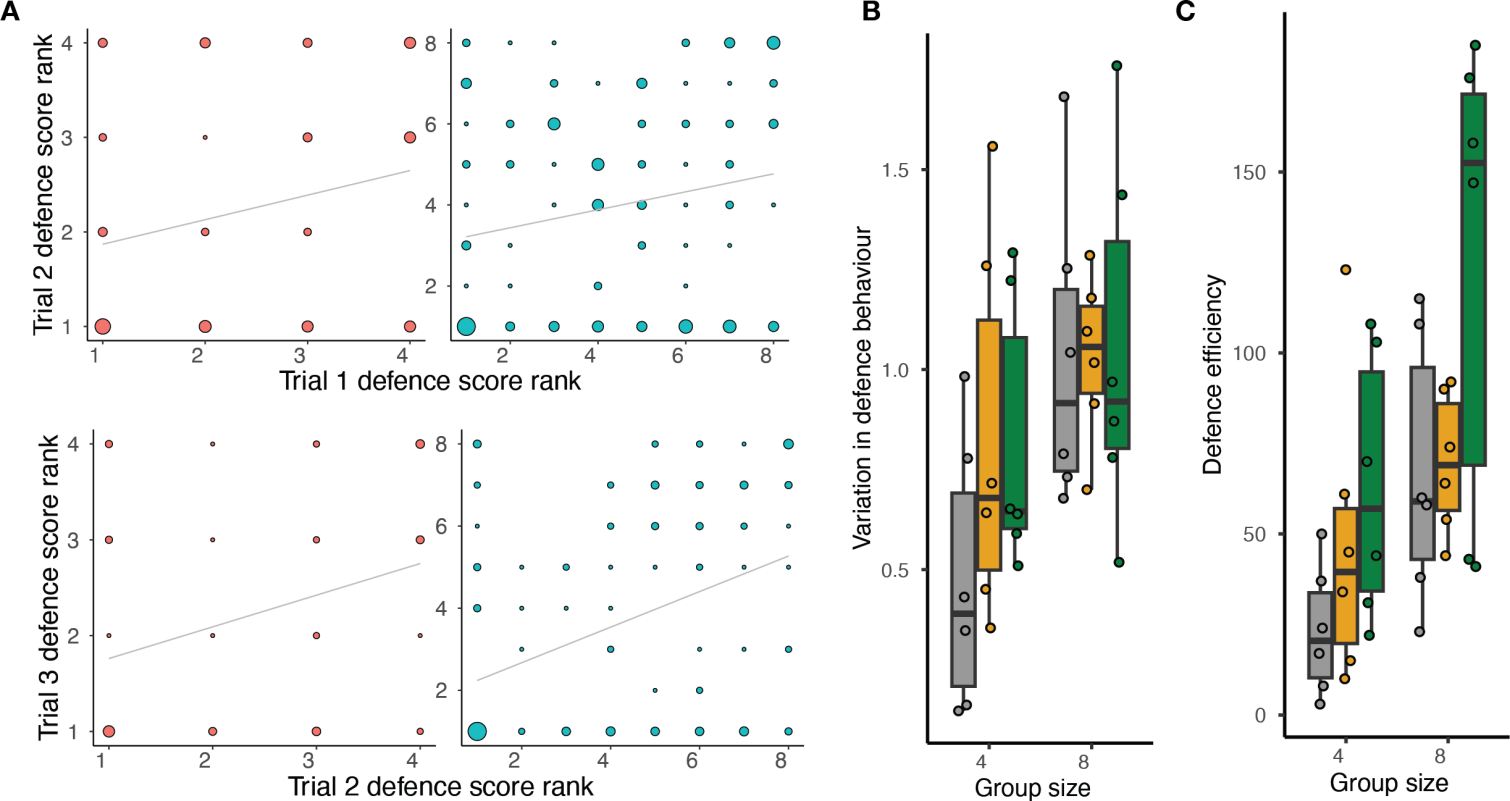
Division of labour in colony defence. (*a*) Behavioural consistency: correlation between ranked defence scores in Trial 1 versus Trial 2 (top) and Trial 2 versus Trial 3 (bottom). Data points represent individuals; data point diameter represents the number of ants with a given rank combination; colours represent group size (red: 4, blue: 8); grey lines: least-squares fit. Data for different brood treatments are pooled. (*b*) Variation in defence behaviour in all trials as a function of group size and brood treatment (grey: no brood, yellow: larvae, green: pupae). Data points represent colonies; boxes indicate the interquartile range (IQR) around the median (thick black line), whiskers represent the highest and lowest values within 1.5 IQR of the upper and lower quartiles, respectively). (*c*) Defence efficiency across all trials as a function of group size and brood treatment (colours as in *b*). Data points represent colonies, boxes indicate the IQR around the median (thick black line) and whiskers represent the highest and lowest values within 1.5 IQR of the upper and lower quartiles, respectively.

We find evidence for DOL in defence behaviour. Within a colony, individual defence scores could range from 0 (an ant encountered the intruder 14 times and never stung) to 4.66 (an ant attempted to sting the intruder in 13 out of 18 encounters for a total duration of 629.25 s) in one trial. Individual defence scores were overall positively correlated across trials, demonstrating individual consistency in defence behaviour (figure 2A; electronic supplementary material, figure S2 and table S5; group size 4: Trial 1 versus Trial 2: *r* = 0.17, *p*-adjusted = 0.195; Trial 2 versus Trial 3: *r* = 0.41, *p-*adjusted = 0.005; group size 8: Trial 1 versus Trial 2: *r* = 0.28, *p*-adjusted = 0.0015; Trial 2 versus Trial 3: *r* = 0.46, *p*-adjusted = 2.21 × 10^−8^). Two lines of evidence show that DOL in defence increased with group size. First, individual consistency in defence behaviour was overall stronger in larger colonies (see above; figure 2A). Second, variation in defence behaviour was higher in larger colonies (figure 2B and table 1, GLMM: d.f. = 1, sum sq. = 0.69, *F* = 5.08, *p* = 0.031) and this effect could not be explained by sampling effects, as shown by the fact that the observed values for small colonies (mean ± s.e.m in colonies of size 4: 0.82 ± 0.08) fell outside the confidence interval generated by resampling from large colonies (95% confidence interval in simulated small colonies resampled from large colonies: (0.92, 1.17)). Finally, DOL (behavioural variation) increased with the mean defence score (d.f. = 1, sum sq. = 11.13, *F* = 82.53, *p* = 3.88 × 10^−14^) but was not affected by trials (d.f. = 2, sum sq. = 0.45, *F* = 1.67, *p* = 0.195) or brood treatment (d.f. = 2, sum sq. = 0.33, *F* = 1.21, *p* = 0.311).

**Table 1.** Effects of group size, brood, trial and mean defence score on behavioural variation.

	d.f.	sum sq.	*F*	*p*
group size	1	0.69	5.08	0.031
brood	2	0.33	1.21	0.311
trial	2	0.45	1.67	0.195
mean defence score	1	11.13	82.53	3.88 × 10^−14^

Colony defence efficiency was influenced by group size (figure 2C; d.f. = 1, sum sq. = 16256, *F* = 10.48, *p* = 0.003) and by the presence and type of brood (d.f. = 2, sum sq. = 64895, *F* = 4.93, *p* = 0.014). Large colonies defended more efficiently than smaller colonies, as expected, since they had more individuals available to participate in the defence. Colonies with pupae had higher defence efficiency than those without brood (figure 2C, Tukey’s *post hoc* test: no brood−pupae, estimate = −48.90, s.e. = 16.10, d.f. = 32, *t* = −3.04, *p* = 0.013), while colonies with larvae did not differ from colonies without brood (no brood−larvae, estimate = −13.80, s.e. = 16.10, d.f. = 32, *t* = −0.85, *p* = 0.672) or from colonies with pupae (larvae−pupae, estimate = −35.20, s.e. = 16.10, d.f. = 32, *t* = −2.19, *p* = 0.089).

Individual baseline exploratory behaviour was positively associated with the number of encounters with the intruder in the first defence trial (GLMM, d.f. = 1, likelihood ratio test (LRT) = 11.28, *p* = 0.001), as expected if clonal raider ants randomly encountered the intruder while patrolling their environment. Both measures of activity were positively correlated (Spearman’s rank correlation test, *r* = 0.86, *p* < 2.2 × 10^−16^) and correlated with exploratory behaviour (proportion of time active and entropy: *r* = 0.97, *p* < 2.2 × 10^–16^; mean duration of active bouts and entropy: *r* = 0.81, *p* < 2.2 × 10^–16^). There was no correlation between individual baseline exploratory behaviour and individual defence score (GLMM, d.f. = 1, LRT = 2.23, *p* = 0.136), or between individual baseline activity and individual defence score (proportion of time active: d.f. = 1, LRT = 3.23, *p* = 0.072; mean duration of active bouts: d.f. = 1, LRT = 0.07, *p* = 0.789). Thus, while exploratory behaviour increased the likelihood of encountering intruders, it did not increase the propensity of an ant to engage in defence upon encounters. Likewise, high activity levels are not associated with a greater propensity to engage in defensive behaviour.

## 4. Discussion

We found DOL in colony defence in *O. biroi*, demonstrated by inter-individual variation and individual consistency in defence behaviour. Although all ants used here had near-identical ages and genotypes and were reared in the same controlled environment, they differed consistently in defence behaviour, suggesting that DOL in colony defence can arise in small, homogeneous social groups. Because we measured the performance of a task in response to a controlled experimental stimulus and corrected for variation in encounter rates with the stimulus, the observed differences in behaviour are likely to reflect individual variation in response thresholds. Variation in response thresholds is one of the theoretically best-studied mechanisms by which DOL can emerge [57,58], but empirically measured variation in response thresholds is still rare. Here, we detect consistent variation in response thresholds that is independent of age or genetic background (as shown by inter-trial consistency in defence scores). As shown in the same species for other behaviours [43], DOL in defence increased with colony size. Although our conclusions are based on the study of a single clonal lineage (B), previous work has shown qualitatively similar patterns of DOL across two commonly used genotypes (A and B) [43], suggesting DOL in colony defence exists in other clonal lineages as well.

The type of brood present in colonies affected defence efficiency, with colonies containing pupae displaying more efficient defence than colonies without brood. This pattern could reflect a context-dependent defence strategy of the colony [59,60]. Pupae used in this study were the product of *ca* 20 days of brood care, a substantial time and resource investment. In addition, late-stage ant pupae have nutritional value for the colony: the moulting fluid they produce is rich in nutrients, hormones and neuroactive substances which are consumed by adults [61]. These factors together may increase the defensive investment in pupae. We did not observe an increase in DOL in colonies with pupae, suggesting that the enhanced defensive investment may be a general colony response rather than the result of increased defensive behaviour in a subset of specialized workers.

We failed to detect a behavioural syndrome linking exploration or activity with aggression as joint manifestations of boldness that correlate across individuals, as has been reported in various species [30–33], including ants [29,35,36]. Here, while ants with higher explorative behaviour encountered intruders more frequently (as expected), they were not more (or less) likely to engage in defence behaviour upon encounter. The discrepancy may stem from the context in which exploratory behaviour and aggression are measured. Previous studies on exploration and aggression in ants often focused on isolated individuals [35,36]. While studying isolated individuals allows the control of social influences and can reveal intrinsic behavioural tendencies, the relationship between exploration and aggression can differ between isolated and social contexts [60]. For instance, ants have been shown to exhibit lower levels of aggression when alone versus in a group owing to the need for colony defence [62] or the presence of social cues [60,63]. In a colony setting, the social environment can amplify individual defence based on the social information individuals perceive (e.g. via alarm pheromones [64,65] or non-nestmate cues [66]). Additionally, past work on social insects involved colonies containing individuals of varying ages, genetic backgrounds and/or morphology. In contrast, we controlled for age and genetic background in *O. biroi*, a monomorphic species, and found no correlation between exploratory and defensive behaviours. This suggests that the behavioural syndrome reported in social insects might stem in part from underlying inter-individual variation in factors such as genotype [67], morphological caste [35] or age [68].

How consistent differences in behaviour arise among genetically identical individuals reared in the same environment is an important, unanswered question. Studies on naturally clonal species (including fish [69], bacteria [70] and insects [71]), as well as animals from isogenic laboratory lines [72,73], and human monozygotic twins [74], consistently show stable inter-individual differences in behaviour, but the developmental and neurological bases of such variation are only starting to be elucidated [72,75]. Social insects are notorious for their extreme developmental phenotypic plasticity. In clonal raider ants, differences in adult behavioural tendencies might arise from small (potentially stochastic) differences in development (e.g. temperature and nutrition at the larval stage). Inherent differences in adult behavioural tendencies may then be plastically amplified by several factors, including group size and composition (via e.g. threshold responses), individual experience [76] or social interactions, to generate stable DOL among group members.

## Data Availability

The data and code for analysis are available from the GitHub repository: https://github.com/lizimai/Li_etal_2025. Supplementary material is available online [77].

## References

[1] West SA , Cooper GA . 2016 Division of labour in microorganisms: an evolutionary perspective. Nat. Rev. Microbiol. **14** , 716–723. (10.1038/nrmicro.2016.111) 27640757

[2] Wilson EO . 1971 The insect societies. Cambridge, MA: Harvard University Press.

[3] Biedermann PHW , Taborsky M . 2011 Larval helpers and age polyethism in ambrosia beetles. Proc. Natl Acad. Sci. USA **108** , 17064–17069. (10.1073/pnas.1107758108) 21969580 PMC3193236

[4] Smith A . 2012 *The wealth of nations* . Ware, UK: Wordsworth Editions.

[5] Bonner JT . 1993 Dividing the labour in cells and societies. Curr. Sci. **64** , 459–466.

[6] Queller DC . 1997 Cooperators since life began. Q. Rev. Biol. **72** , 184–188. (10.1086/419766)

[7] Szathmáry E , Maynard Smith J . 1995 The major evolutionary transitions. Nature **374** , 227–232. (10.1038/374227a0) 7885442

[8] Bourke AFG . 2011 Principles of social evolution. Oxford, UK: Oxford University Press.

[9] Zöttl M , Vullioud P , Mendonça R , Torrents Ticó M , Gaynor D , Mitchell A , Clutton-Brock T . 2016 Differences in cooperative behavior among Damaraland mole rats are consequences of an age-related polyethism. Proc. Natl Acad. Sci. USA **113** , 10382–10387. (10.1073/pnas.1607885113) 27588902 PMC5027444

[10] Julian GE , Fewell JH . 2004 Genetic variation and task specialization in the desert leaf-cutter ant, Acromyrmex versicolor. Anim. Behav. **68** , 1–8. (10.1016/j.anbehav.2003.06.023) 10458895

[11] Liu M , West SA , Cooper GA . 2021 Relatedness and the evolution of mechanisms to divide labor in microorganisms. Ecol. Evol. **11** , 14475–14489. (10.1002/ece3.8067) 34765120 PMC8571581

[12] Robinson GE . 1992 Regulation of division of labor in insect societies. Annu. Rev. Entomol. **37** , 637–665. (10.1146/annurev.en.37.010192.003225) 1539941

[13] Wilson EO . 1990 Success and dominance in ecosystems: the case of the social insects. Oldendorf/Luhe, Germany: Ecology Institute.

[14] Schultheiss P , Nooten SS , Wang R , Wong MKL , Brassard F , Guénard B . 2022 The abundance, biomass, and distribution of ants on Earth. Proc. Natl Acad. Sci. USA **119** , e2201550119. (10.1073/pnas.2201550119) 36122199 PMC9546634

[15] Wilson EO . 2012 The social conquest of Earth. New York, NY: WW Norton & Company.

[16] Traniello JFA , Rosengaus RB . 1997 Ecology, evolution and division of labour in social insects. Anim. Behav. **53** , 209–213. (10.1006/anbe.1996.0289)

[17] Hölldobler B , Wilson EO . 1990 The ants. Cambridge, MA: Harvard University Press.

[18] Wheeler WM . 1911 The ant-colony as an organism. J. Morphol. **22** , 307–325.

[19] Nouvian M , Breed MD . 2021 Colony defense by social insects. In Encyclopedia of social insects (ed. K Starr ), pp. 230–240. Cham, Switzerland: Springer International Publishing.

[20] Barth MB , Kellner K , Heinze J . 2010 The police are not the army: context-dependent aggressiveness in a clonal ant. Biol. Lett. **6** , 329–332. (10.1098/rsbl.2009.0849) 20071394 PMC2880046

[21] Abbot P . 2022 Defense in social insects: diversity, division of labor, and evolution. Annu. Rev. Entomol. **67** , 407–436. (10.1146/annurev-ento-082521-072638) 34995089

[22] Cremer S , Sixt M . 2009 Analogies in the evolution of individual and social immunity. Phil. Trans. R. Soc. B **364** , 20080166. (10.1098/rstb.2008.0166) PMC2666697 18926974

[23] Esponda F , Gordon DM . 2015 Distributed nestmate recognition in ants. Proc. R. Soc. B **282** , 20142838. (10.1098/rspb.2014.2838) PMC4426612 25833853

[24] Engel MS , Barden P , Riccio ML , Grimaldi DA . 2016 Morphologically specialized termite castes and advanced sociality in the early Cretaceous. Curr. Biol. **26** , 522–530. (10.1016/j.cub.2015.12.061) 26877085

[25] Jaffé R , Kronauer DJC , Kraus FB , Boomsma JJ , Moritz RFA . 2007 Worker caste determination in the army ant Eciton burchellii. Biol. Lett. **3** , 513–516. (10.1098/rsbl.2007.0257) 17638672 PMC2391184

[26] Grüter C , Menezes C , Imperatriz-Fonseca VL , Ratnieks FLW . 2012 A morphologically specialized soldier caste improves colony defense in a neotropical eusocial bee. Proc. Natl Acad. Sci. USA **109** , 1182–1186. (10.1073/pnas.1113398109) 22232688 PMC3268333

[27] Oster GF , Wilson EO . 1978 Caste and ecology in the social insects. Princeton, NJ: Princeton University Press. 740003

[28] Yanagihara S , Suehiro W , Mitaka Y , Matsuura K . 2018 Age-based soldier polyethism: old termite soldiers take more risks than young soldiers. Biol. Lett. **14** , 20180025. (10.1098/rsbl.2018.0025) 29514993 PMC5897614

[29] Jandt JM , Bengston S , Pinter‐Wollman N , Pruitt JN , Raine NE , Dornhaus A , Sih A . 2014 Behavioural syndromes and social insects: personality at multiple levels. Biol. Rev. **89** , 48–67. (10.1111/brv.12042) 23672739

[30] Bergmüller R , Taborsky M . 2007 Adaptive behavioural syndromes due to strategic niche specialization. BMC Ecol. **7** , 12. (10.1186/1472-6785-7-12) 17935618 PMC2104524

[31] Thys B , Pinxten R , Raap T , De Meester G , Rivera-Gutierrez HF , Eens M . 2017 The female perspective of personality in a wild songbird: repeatable aggressiveness relates to exploration behaviour. Scient. Rep. **7** , 7656. (10.1038/s41598-017-08001-1) PMC5550452 28794486

[32] Garamszegi LZ , Eens M , Török J . 2009 Behavioural syndromes and trappability in free-living collared flycatchers, Ficedula albicollis. Anim. Behav. **77** , 803–812. (10.1016/j.anbehav.2008.12.012)

[33] Sih A , Del Giudice M . 2012 Linking behavioural syndromes and cognition: a behavioural ecology perspective. Phil. Trans. R. Soc. B **367** , 2762–2772. (10.1098/rstb.2012.0216) 22927575 PMC3427552

[34] Verbeek MEM , Boon A , Drent PJ . 1996 Exploration, aggressive behaviour and dominance in pair-wise confrontations of juvenile male great tits. Behaviour **133** , 945–963. (10.1163/156853996x00314)

[35] Chapman BB , Thain H , Coughlin J , Hughes WOH . 2011 Behavioural syndromes at multiple scales in Myrmica ants. Anim. Behav. **82** , 391–397. (10.1016/j.anbehav.2011.05.019)

[36] Modlmeier AP , Liebmann JE , Foitzik S . 2012 Diverse societies are more productive: a lesson from ants. Proc. R. Soc. B **279** , 2142–2150. (10.1098/rspb.2011.2376) PMC3321703 22279166

[37] Blight O , Albet Díaz-Mariblanca G , Cerdá X , Boulay R . 2016 A proactive–reactive syndrome affects group success in an ant species. Behav. Ecol. **27** , 118–125. (10.1093/beheco/arv127)

[38] Walton A , Toth AL . 2016 Variation in individual worker honey bee behavior shows hallmarks of personality. Behav. Ecol. Sociobiol. **70** , 999–1010. (10.1007/s00265-016-2084-4)

[39] Wilson EO . 1968 The ergonomics of caste in the social insects. Am. Nat. **102** , 41–66. (10.1086/282522)

[40] Blatrix R . 2000 Task allocation depends on matriline in the ponerine ant Gnamptogenys striatula Mayr. J. Insect Behav. **47** , 193–197. (10.1007/PL00001701)

[41] Lucas C , Ben-Shahar Y . 2021 The foraging gene as a modulator of division of labour in social insects. J. Neurogenet. **35** , 168–178. (10.1080/01677063.2021.1940173) 34151702

[42] Ravary F , Jaisson P . 2002 The reproductive cycle of thelytokous colonies of Cerapachys biroi Forel (Formicidae, Cerapachyinae). Insectes Sociaux **49** , 114–119. (10.1007/s00040-002-8288-9)

[43] Ulrich Y , Saragosti J , Tokita CK , Tarnita CE , Kronauer DJC . 2018 Fitness benefits and emergent division of labour at the onset of group living. Nature **560** , 635–638. (10.1038/s41586-018-0422-6) 30135576 PMC6121774

[44] Fetter-Pruneda I *et al* . 2021 An oxytocin/vasopressin-related neuropeptide modulates social foraging behavior in the clonal raider ant. PLoS Biol. **19** , e3001305. (10.1371/journal.pbio.3001305) 34191794 PMC8244912

[45] Trible W , McKenzie SK , Kronauer DJC . 2020 Globally invasive populations of the clonal raider ant are derived from Bangladesh. Biol. Lett. **16** , 20200105. (10.1098/rsbl.2020.0105) 32544382 PMC7336853

[46] Chandra V , Gal A , Kronauer DJC . 2021 Colony expansions underlie the evolution of army ant mass raiding. Proc. Natl Acad. Sci. USA **118** , e2026534118. (10.1073/pnas.2026534118) 34035172 PMC8179172

[47] Roulston TH , Buczkowski G , Silverman J . 2003 Nestmate discrimination in ants: effect of bioassay on aggressive behavior. Insectes Sociaux **50** , 151–159. (10.1007/s00040-003-0624-1)

[48] Modlmeier AP , Foitzik S . 2011 Productivity increases with variation in aggression among group members in Temnothorax ants. Behav. Ecol. **22** , 1026–1032. (10.1093/beheco/arr086)

[49] Gal A , Saragosti J , Kronauer DJC . 2020 anTraX, a software package for high-throughput video tracking of color-tagged insects. eLife **9** , e58145. (10.7554/eLife.58145) 33211008 PMC7676868

[50] Jud SL , Knebel D , Ulrich Y . 2022 Intergenerational genotypic interactions drive collective behavioural cycles in a social insect. Proc. R. Soc. B **289** , 20221273. (10.1098/rspb.2022.1273) PMC9627708 36321497

[51] Friard O , Gamba M . 2016 BORIS: a free, versatile open‐source event‐logging software for video/audio coding and live observations. Methods Ecol. Evol. **7** , 1325–1330. (10.1111/2041-210x.12584)

[52] Kronauer DJC , Tsuji K , Pierce NE , Keller L . 2013 Non-nest mate discrimination and clonal colony structure in the parthenogenetic ant Cerapachys biroi. Behav. Ecol. **24** , 617–622. (10.1093/beheco/ars227)

[53] R Core Team . 2020 R: a language and environment for statistical computing. Vienna, Austria: R Foundation for Statistical Computing. See http://www.R-project.org/.

[54] Hanisch PE , Hanisch ER , Blanco V , Tubaro PL , Suarez AV . 2023 Spatial fidelity and uniform exploration in the foraging behaviour of a giant predatory ant. Anim. Behav. **203** , 63–73. (10.1016/j.anbehav.2023.06.009)

[55] Suarez AV , Tsutsui DN , Holway DA , Case TJ . 1999 Behavioral and genetic differentiation between native and introduced populations of the Argentine ant. Biol. Invasions **1** , 43–53. (10.1023/A:1010038413690)

[56] Gu G , Meng Y , Tan K , Dong S , Nieh JC . 2021 Lethality of honey bee stings to heavily armored hornets. Biology **10** , 484. (10.3390/biology10060484) 34072577 PMC8229339

[57] Beshers SN , Fewell JH . 2001 Models of division of labor in social insects. Annu. Rev. Entomol. **46** , 413–440. (10.1146/annurev.ento.46.1.413) 11112175

[58] Bonabeau E , Theraulaz G . 1999 Role and variability of response thresholds in the regulation of division of labor in insect societies. In Information processing in social insects (eds C Detrain , JL Deneubourg , JM Pasteels ), pp. 141–163. Basel, Switzerland: Birkhäuser Basel. (10.1007/978-3-0348-8739-7_8)

[59] Cole BJ . 1988 Escalation of aggression in Leptothorax ants. Insectes Sociaux **35** , 198–205. (10.1007/bf02223933)

[60] Sakata H , Katayama N . 2001 Ant defence system: a mechanism organizing individual responses into efficient collective behavior. Ecol. Res. **16** , 395–403. (10.1046/j.1440-1703.2001.00404.x)

[61] Snir O , Alwaseem H , Heissel S , Sharma A , Valdés-Rodríguez S , Carroll TS , Jiang CS , Razzauti J , Kronauer DJC . 2022 The pupal moulting fluid has evolved social functions in ants. Nature **612** , 488–494. (10.1038/s41586-022-05480-9) 36450990 PMC9750870

[62] Uematsu J , Hayashi M , Shimoji H , Laurent Salazar MO , Tsuji K . 2019 Context-dependent aggression toward non-nestmates in the ant Diacamma sp. from Japan. J. Ethol. **37** , 259–264. (10.1007/s10164-019-00611-8)

[63] Kleineidam CJ , Heeb EL , Neupert S . 2017 Social interactions promote adaptive resource defense in ants. PLoS One **12** , e0183872. (10.1371/journal.pone.0183872) 28910322 PMC5598949

[64] Guo X , Lin MR , Azizi A , Saldyt LP , Kang Y , Pavlic TP , Fewell JH . 2022 Decoding alarm signal propagation of seed-harvester ants using automated movement tracking and supervised machine learning. Proc. R. Soc. B **289** , 20212176. (10.1098/rspb.2021.2176) PMC8790334 35078355

[65] López-Incera A , Nouvian M , Ried K , Müller T , Briegel HJ . 2021 Honeybee communication during collective defence is shaped by predation. BMC Biol. **19** , 106. (10.1186/s12915-021-01028-x) 34030690 PMC8147350

[66] Bey M , Endermann R , Raudies C , Steinle J , Nehring V . 2025 Associative learning of non-nestmate cues improves enemy recognition in ants. Curr. Biol. **35** , 407–412. (10.1016/j.cub.2024.11.054) 39742675

[67] Alaux C *et al* . 2009 Honey bee aggression supports a link between gene regulation and behavioral evolution. Proc. Natl Acad. Sci. USA **106** , 15400–15405. (10.1073/pnas.0907043106) 19706434 PMC2730357

[68] Judd TM . 2000 Division of labour in colony defence against vertebrate predators by the social wasp Polistes fuscatus. Anim. Behav. **60** , 55–61. (10.1006/anbe.2000.1449) 10924203

[69] Laskowski KL , Bierbach D , Jolles JW , Doran C , Wolf M . 2022 The emergence and development of behavioral individuality in clonal fish. Nat. Commun. **13** , 6419. (10.1038/s41467-022-34113-y) 36307437 PMC9616841

[70] Pleška M , Jordan D , Frentz Z , Xue B , Leibler S . 2021 Nongenetic individuality, changeability, and inheritance in bacterial behavior. Proc. Natl Acad. Sci. USA **118** , e2023322118. (10.1073/pnas.2023322118) 33753503 PMC8020789

[71] Schuett W , Dall SRX , Baeumer J , Kloesener MH , Nakagawa S , Beinlich F , Eggers T . 2011 Personality variation in a clonal insect: the pea aphid, Acyrthosiphon pisum. Dev. Psychobiol. **53** , 631–640. (10.1002/dev.20538) 21365642

[72] Freund J , Brandmaier AM , Lewejohann L , Kirste I , Kritzler M , Krüger A , Sachser N , Lindenberger U , Kempermann G . 2013 Emergence of individuality in genetically identical mice. Science **340** , 756–759. (10.1126/science.1235294) 23661762

[73] de Bivort B *et al* . 2022 Precise quantification of behavioral individuality from 80 million decisions across 183,000 flies. Front. Behav. Neurosci. **16** , 836626. (10.3389/fnbeh.2022.836626) 35692381 PMC9178272

[74] Johnson W , Turkheimer E , Gottesman II , Bouchard Jr TJ . 2009 Beyond heritability. Curr. Direct. Psychol. Sci. **18** , 217–220. (10.1111/j.1467-8721.2009.01639.x) PMC2899491 20625474

[75] Honegger KS , Smith MAY , Churgin MA , Turner GC , de Bivort BL . 2020 Idiosyncratic neural coding and neuromodulation of olfactory individuality in Drosophila. Proc. Natl Acad. Sci. USA **117** , 23292–23297. (10.1073/pnas.1901623116) 31455738 PMC7519279

[76] Ravary F , Lecoutey E , Kaminski G , Châline N , Jaisson P . 2007 Individual experience alone can generate lasting division of labor in ants. Curr. Biol. **17** , 1308–1312. (10.1016/j.cub.2007.06.047) 17629482

[77] Li Z , Wang Q , Knebel D , Veit D , Ulrich Y . 2025 Supplementary material from: Division of labour in colony defence in a clonal ant. Figshare. (10.1101/2024.02.16.580644) PMC11969388 40109105

